# Revising ethical guidance for the evaluation of programmes and interventions not initiated by researchers

**DOI:** 10.1136/medethics-2018-105263

**Published:** 2019-09-03

**Authors:** Samuel I Watson, Mary Dixon-Woods, Celia A Taylor, Emily B Wroe, Elizabeth L Dunbar, Peter J Chilton, Richard J Lilford

**Affiliations:** 1 Warwick Medical School, University of Warwick, Coventry, UK; 2 THIS Institute, University of Cambridge, Cambridge, UK; 3 Partners In Health, Boston, Massachusetts, USA; 4 Warwick Business School, University of Warwick, Coventry, UK

**Keywords:** Research ethics, clinical trials, policy guidelines

## Abstract

Public health and service delivery programmes, interventions and policies (collectively, ‘programmes’) are typically developed and implemented for the primary purpose of effecting change rather than generating knowledge. Nonetheless, evaluations of these programmes may produce valuable learning that helps determine effectiveness and costs as well as informing design and implementation of future programmes. Such studies might be termed ‘opportunistic evaluations’, since they are responsive to emergent opportunities rather than being studies of interventions that are initiated or designed by researchers. However, current ethical guidance and registration procedures make little allowance for scenarios where researchers have played no role in the development or implementation of a programme, but nevertheless plan to conduct a prospective evaluation. We explore the limitations of the guidance and procedures with respect to opportunistic evaluations, providing a number of examples. We propose that one key missing distinction in current guidance is moral responsibility: researchers can only be held accountable for those aspects of a study over which they have control. We argue that requiring researchers to justify an intervention, programme or policy that would occur regardless of their involvement prevents or hinders research in the public interest without providing any further protections to research participants. We recommend that trial consent and ethics procedures allow for a clear separation of responsibilities for the intervention and the evaluation.

## Introduction

In this article, we focus on the ethics of a distinctive type of study: evaluations of programmes, initiatives, policies and interventions that would occur *whether or not* any research activity was taking place. That is to say, in these cases the research is not the cause, in a counterfactual sense, of the intervention being applied to human subjects. The *primary goal*, from the perspective of the leaders and the organisations initiating and implementing the activities, which we will collectively term ‘programmes’, is to make change and improvement, not to produce knowledge. The impetus for the activity may arise external to organisations (eg, in response to policy or contractual changes, opportunities to work in national programmes, and so on), or internally (eg, in response to discovery of a problem or regulatory finding, through service improvement agendas, and so on).

These kinds of programmes are very common. They range from large-scale service reconfigurations and pathway redesign through to more local changes in appointment systems, supply chains and operating theatre scheduling. Current UK examples include the Getting It Right First Time programme, which seeks to reduce unwarranted clinical variations with ~£60 million funding,^1^ and the roll-out of the National Early Warning Score system^2^ to improve detection and response to deteriorating patients in hospitals. In these cases, healthcare organisations will be initiating the programmes anyway, but opportunistic evaluations conducted alongside them have the potential to advance knowledge about programme design, implementation, mechanisms of change and outcomes.

Thus, though researchers may have no role whatsoever in the development and implementation of these programmes, they may have a very important role in evaluating them. In this article, we propose that current ethical guidelines for the conduct of research are a barrier to prospective evaluations of programmes that are independent of researchers. Specifically, guidelines act as a hindrance because they assume that researchers have responsibility for factors over which, in reality, they have no control. In particular, we shall be arguing that some of the principles outlined in the Ottawa statement on cluster randomised trials^3^ should be relaxed when researchers evaluate an intervention that will occur regardless of any study. We propose that, as a basic principle, researchers cannot be held responsible for programmes that they are not accountable for, that are not attributable to their involvement and that they have no control over.

## Opportunistic evaluations

Done well and systematically, evaluative studies that are conducted alongside programmes that are happening anyway serve the interests of patients, the public and the healthcare system by producing systematic knowledge about the fate and impact of improvement and change programmes.^4^ We term these studies ‘opportunistic evaluations’, and define them as follows:

Opportunistic evaluations study programmes, initiatives, policies, and interventions that would occur whether or not there were any concurrent, coincident, or otherwise related research activities designed to produce knowledge from the programme. Opportunistic evaluations are systematic studies that make use of naturally arising opportunities to study the effects and costs of those programmes and how they work.

Opportunistic evaluations can make invaluable contributions to advancing knowledge about programme design, implementation, mechanisms of change and outcomes. For example, they can assist with determining effectiveness, identifying the influences on effectiveness, characterise the mechanisms of action, support replication and scaling of successful programmes, and reduce waste and costs associated with unsuccessful programmes by demonstrating what should not be supported in the future. It is especially important that initiatives and programmes be evaluated if they are novel, expensive, have the potential for significant impact, or there are plausible reasons to evaluate them in a new context.^5 6^ In such situations, there is a strong public interest argument for evaluation.

The Ottawa statement on the ethical design and conduct of cluster randomised trials,^3^ however, makes little of acknowledgement of a scenario where the researchers have not initiated the programme. For example, it draws on the principle of clinical equipoise to require researchers to ‘ensure that the study intervention is adequately justified.’ While this might be an appropriate safeguard when researchers design interventions for purposes of generating new knowledge, it is unduly restrictive when the intervention is wholly owned in the service. Indeed, the public interest in evaluating an intervention may arise because there is a *lack* of equipoise. For example, in the USA the Centers for Medicare and Medicaid Services recently invited states to establish work requirements for Medicaid benefits. A number of physician organisations and other groups have argued that these requirements are likely to lead to financial and health harms for patients.^7^ Prospectively examining the impact of this policy is essential to fully informing future debate.

The point that interventions may not be initiated by researchers but nonetheless warrant study is exemplified by the demand for evaluation from those who have designed, commissioned or implemented programmes and policies and the usefulness of the evidence such evaluations generate. As an example, 24 hospitals across the UK participated in the Safer Patients Programme, which was commissioned by the Health Foundation, a UK charitable foundation. Those hospitals took part in the programme in order to improve their systems, practices and culture—not to produce knowledge. An evaluation of the programme did not change anything that the organisations were going to do anyway, but it did show that, on average, those organisations did not improve more than organisations that were not participating in the programme, thus producing very useful learning about whether the programme was a suitable candidate for investment by the National Health Service (NHS).^8 9^

Another example is the evaluation of the reconfiguration of stroke services in two major UK cities: London and Manchester. The evaluation again was wrapped around a programme that was happening anyway, and produced important learning about centralised models, showing that they can reduce mortality and length of stay.^10^ The evaluation of the Mexican Progresa/Oportunidades programme, which included conditional cash transfers for certain health and educational outcomes, provides another illustration. Randomisation was built into the government’s implementation of the programme to enable experimental evaluation without affecting the policy itself or the eventual recipients. A great deal of valuable research has been published on the basis of this roll-out and subsequent follow-up studies on the effects of conditional cash transfer programmes, which now over 60 countries implement.^11^

A defining feature of opportunistic evaluations, in these kinds of scenarios, is that those studying the programme do not control the change under study: the evaluators do not conceive of, initiate, deliver, or affect the programme of interventions or other changes. Yet current governance and ethics requirements may be problematic or ill suited to the goals either of evaluation or to organisations-seeking improvement, since they typically assume that the evaluator is controlling many more aspects of the programme than is usually the case.

## Study designs for opportunistic evaluations

In identifying the challenges associated with opportunistic evaluations, it is useful first to expand on what constitutes an ‘opportunistic evaluation’. When a programme is designed and led by researchers, even if implemented by policymakers, the researcher bears responsibility, and the normal rules of ethical engagement should apply. For instance, a service innovation to test an intervention to provide more patient-centred care^12^ clearly falls into this category, since the participating professionals and organisations would not have implemented it without a research project. Our concern is not with these researcher-led programmes, but where the researcher has not designed or initiated the programme. The distinction has analogies with the distinction between prospective and retrospective studies.

With a retrospective study of a programme not initiated by a researcher, the programme lies in the past and the researcher cannot be assumed to have responsibility for it. Nevertheless, these studies can take advantage of the opportunities afforded by how programmes were rolled out. For example, the lottery mechanism used to allocate limited sign-up opportunities for Medicaid in Oregon, USA, afforded researchers an opportunity to examine the effects of healthcare coverage that took advantage of a randomisation process that had been deployed for reasons other than research, and over which the researchers had no control.^13 14^ Likewise, allocation of permits by lottery to allow Fijians to emigrate to New Zealand allowed researchers to study the effects of migration on health and well-being many years later.^15 16^ Other studies may take the form of natural experiments: observational studies where a researcher ‘can make a credible claim that the assignment of the nonexperimental subjects to treatment and control conditions is ‘as if’ random.’^17^ For instance, a study in Canada of the impact of a pay-for-performance scheme, where some but not all of the physicians were exposed to the incentives, allowed a quasiexperimental evaluation where no aspect of the assignment of the physicians to the programme was controlled by the researchers.^18^

Retrospective evaluations of programmes that have already occurred clearly absolve researchers from responsibility for the programme itself, but they do not absolve researchers of ethical responsibility for the research activities they conduct. As for all studies, researchers must obtain ethics committee approval for all the things that they plan to do in their roles as researchers: data collection, analysis, protection of rights of participants, and so on. They have unassailable duties to protect anonymity, properly interpret data and avoid ‘over-claiming’. They must not fabricate or plagiarise data, and should place their work in the public domain.

These obligations apply equally to prospective studies of programmes that are not researcher initiated. Prospective study designs are often highly beneficial to science and learning. They may, for example, enable hypotheses to be specified a priori, thus mitigating the risks of data-driven comparisons. Evaluators can support augmentation of the information to be collected—for example, qualitative data to explicate implementation fidelity, or surveys of participants’ views, thus maximising the learning from the programme. A statistical analysis plan can also be specified, thus enhancing transparency and rigour. However, a number of ambiguities complicate clarity about the ethical duties of the evaluators in prospective opportunistic evaluations, which vary somewhat across two types of study.

In the first, the evaluator has no influence on the programme at all, but uses available data and the design of the programme to mount a study prospectively. As an example, an opportunistic evaluation of the 7 Day Services policy implemented across the NHS in England and Wales, which aimed to increase specialist availability in hospitals at the weekend, is examining a policy that was designed and implemented by the Department of Health and NHS England independently of the evaluation. The evaluation involves a review of patient case notes from before and after the policy implementation alongside qualitative studies during the implementation itself.^19^

The second study type is one where the programme is not initiated by researchers but they help to design the implementation (not the intervention) in a way that facilitates evaluation, for example, by proposing to programme leaders that study design such as wait-list, stepped-wedge or cluster randomised designs be adopted in situations where use of such strategies is consistent with the goals of the programme. This approach is often true when a programme cannot be rolled out to all possible sites at once, and much scientific value can be obtained by the order of roll-out to be determined at random.^20^ Importantly, though, this still meets our definition of an opportunistic evaluation in that the intervention would still occur without the involvement of researchers. Such was the case with the aforementioned Progresa/Oportunidades programme, a poverty alleviation programme in Mexico, which incorporated a randomised implementation to permit rigorous evaluation.^11^ Another example was the evaluation of the Matching Michigan programme in the English NHS, which incorporated a stepped (although non-random) roll-out.^21^

Very often, the most convenient and robust prospective study design for programmes involves allocation of interventions at the cluster level (eg, village, school, hospital ward) rather than the individual level. In the current literature, including the Ottawa declaration, cluster randomised studies are considered to pose distinctive ethical issues.^22^ However, many of these issues are not relevant to opportunistic evaluations, since there is little that is ethically fraught about randomising roll-out in a situation where every site is going to receive the programme anyway, where they cannot all receive it at once and where the role of the researchers is to support roll-out in a manner that optimises learning.

## Ethical debates about cluster randomisation

The most influential guidance on the ethics of cluster randomisation is found in the *Ottawa Statement on the design and conduct of cluster randomised trials*.^3^ Its emphases are largely consistent with most current codifications of ethical practice,^23–26^ yet they make for a remarkably poor fit with the realities of opportunistic evaluations.

One evident problem is that the Ottawa statement calls on the researcher to justify the rationale for the intervention and demonstrate equipoise.^3^ But, for opportunistic evaluations, the researchers may not be well placed to supply the rationale (after all, it is not their choice), much less justify it, especially since their role may be one of surfacing the rationale and assessing its soundness.^27^

The Ottawa declaration also holds that the researcher is responsible for obtaining informed consent for the intervention from participants or if the intervention has minimal harm, obtain a waiver of consent. However, it should not be assumed that planned programmes are free of an a priori expectation of harm from the intervention. Consider again the Medicaid work requirements policy discussed above. A waiver of consent would not be granted for this intervention; indeed, it is unlikely participants would even consent to the intervention, and yet it would still occur regardless of the consent process for research. While the researcher can properly be held responsible for obtaining informed consent for any data collection for research purposes, this responsibility should not extend to the intervention itself, since it is implemented under a policy or leadership mandate—it would be strange indeed for a researcher to seek consent from members of a cluster for an intervention that the policymaker had decided to introduce regardless of any evaluation.

It has also been argued that researchers must justify the choice of control condition in cluster studies.^3 22^ While it is correct that the researcher, given a choice of possible controls, should select the controls to maximise their scientific value, the researcher cannot be held responsible for the fact that controls have not received the intervention, since it is the policymaker, not the researcher, who decides where and when to intervene (and where not to do so). As such, controls may not meet the high standard of a trial where the researcher has full control of all aspects of the intervention. Insisting that the intervention must receive ethical approval simply because it is part of an evaluative study does not solve the problem. It would be perverse if, by agreeing to an evaluation of an intervention, a government minister claiming a democratic mandate or a hospital chief acting on behalf of their board, then had to subject the intervention to a research ethics committee, when they could otherwise proceed unhindered: an example of the adage that ‘you can do anything you like, as long as you promise not to learn from it.’

The consequences of the poor alignment between ethical recommendations and the specificities of improvement evaluations are highly practical and far reaching. For instance, the assumption that the researcher controls any prospectively evaluated intervention is so strongly entrenched that it is difficult to register an evaluation of a policymaker’s intervention on ClinicalTrials.gov, the first, largest and most widely used clinical trials registry.^28^ If individuals are not ‘assigned by an investigator… to receive specific interventions,’ then the study must be classified as observational, according to their guidelines. Moreover, having entered ‘observational’ the study cannot then be classified as prospective, but must be classified as retrospective in order to be accepted by the system. Thus, a prospective stepped-wedge randomised controlled trial in which researcher involvement with the intervention and its implementation is limited only to randomising the order of units in the roll-out must be classified as a retrospective, observational study. The implication is that an investigator cannot mount a prospective study of an intervention implemented by a policymaker or programme leader. The pre-registration of a trial is an essential component to ensuring its validity. The impossibility of registering the type of study we describe here on the largest trials registry is indicative of the lack of consideration given to the ‘grey area’ of opportunistic evaluation and further serves as another hindrance to conducting valid studies with appropriate oversight.

Negotiating through these ambiguities leads to delays that may be highly consequential for real-time improvement programmes, to queries at organisation level about cost recovery (because of the difficulties of distinguishing programme activities from evaluation activity) and to inappropriate requirements for local collaborators/principal investigators. Before we suggest how this difficulty might be avoided, we need to examine the ethical principle on which we shall rely.

## Ethics of opportunistic evaluations

Many of the problems we describe above are not resolved by classifying opportunistic evaluations as audit, quality improvement, or service evaluation, which the UK Health Research Authority specifies as comprising projects which are ‘designed and conducted solely to define or judge current care.’^29^ Such projects may not have sufficiently robust governance or ethical oversight and are subject to widely varying local arrangements. A further important problem is that where opportunistic evaluations are not classified as research, the ability to publish findings and accumulate knowledge is undermined, and the credibility and impact of the evaluation on practice and policy is weakened. Yet a significant percentage of evaluations are classified as ‘service evaluations’: Chen and Fawcett note that South East Scotland Research Ethics Service estimates it gave advice on 1300 studies between 2010 and 2015 of which 70% were classified as ‘not research’.^30^ This is not to say that *all* studies should be classified as research, merely that there is strong reason to believe that too many studies are classified as non-research in order to avoid certain regulatory or ethical barriers.

Once it is determined that an opportunistic evaluation counts as research, our basic principle is that a person can only assume (moral) responsibility, or be held accountable, for something that occurs as a result of their action (or absence of action).^31^ The responsibility for an intervention therefore lies with those whose action led to the intervention being designed and implemented. Clinical innovators, such as clinicians or pharmaceutical companies, are held accountable through strict ethical scrutiny, which grants authority to intervene.^22 32^ The authority of those who initiate improvement and change programmes is different, deriving, for example, from democratic mandate. In contrast, the researcher can only bear responsibility for, and hence requires scrutiny for, an intervention when she has participated in designing or implementing it. This position derives from the Kantian principle that ‘ought implies can’^33^: to be held accountable, a moral agent must know of the standards she is expected to meet, be charged with responsibility for meeting those standards, and have sufficient autonomy and capacity in her choice of actions, and access to resources, to be able to comply. When a researcher is unable to influence programme design or implementation, she is not accountable for it.

We propose that, for opportunistic evaluations, the policymaker/programme leader is responsible for the design and implementation of the programme: the evaluator is not (she has other responsibilities, but not for the programme). A conceptual litmus test would be to determine what would occur in the counterfactual scenario of no evaluation taking place. If, in the absence of evaluation, the implementation proceeds in the same way, it follows that the researcher should not be the responsible agent when it comes to programmes.

This is, of course, not to argue that a researcher *qua* researcher can simply abandon their obligations *qua* morally responsible agent. One challenge arises, for example, in circumstances when it might be morally indefensible for researchers to conduct an evaluation at all—for example, when a programme is highly likely or certain to cause significant or severe harm—because of the risk that the evaluation of a programme might vicariously endorse an immoral act. The question of whether a researcher ought to participate in a given evaluation is one that should be judged on a case-by-case basis, given that an evaluation might be ethical even if a programme is not (since the evaluation may, for instance, evidence harm that would otherwise remain concealed). The key point, for purposes of our argument, is that the current ethical guidance for cluster trials requires that the evaluation *and* intervention be justified on grounds of equipoise, minimal harm, and so forth, which would rule out many potentially beneficial evaluations of programmes. Given evidence that implementation of an untested policy based on intuition about what works may be less likely to invite objection from the public than rigorous evaluation of two or more otherwise unobjectionable policies,^34^ the risk is that policymakers or others continue to see imposing a programme on the whole population without evaluation as less problematic (and less trouble) than introducing it for half, and learning in the process. To safeguard the public interest, a new approach to the ethics of opportunistic evaluations is needed. We propose that the approvals necessary for a *researcher* should be limited only to factors over which the researcher has control. We provide an example of the prospective evaluation of an intervention deployed by policymakers in box 1.

Box 1 **An integrated community health worker programme**

Community health workers (CHW) are lay community members trained to provide advice and support ongoing care for a range of conditions, and are an integral part of healthcare delivery in low/middle-income countries. One non-governmental organisation, known as Partners In Health (PIH), provides healthcare infrastructure, programmes and personnel in Neno District in Malawi.

In conjunction with PIH, our research team are evaluating the effects of a programme that changes the organisation, role and activities of the CHWs.^35^ The roll-out of the new CHW model was already planned to be staggered over time between six study sites (clusters) selected by PIH to ensure training feasibility. To avoid the perception of catchment area favouritism, PIH decided to randomise the order in which clusters would receive the intervention, and the research conducted the randomisation. The research team also conducts the statistical analyses using data routinely collected by the CHWs, the health centres and the Ministry of Health. In collaboration with PIH, the research team designed what data should be analysed and how. PIH sought and received ethical approval to implement the change to CHW provision and permissions to evaluate its effects using routinely collected data. The ethical responsibilities of the researchers comprised data sharing and management, and justification of the analyses, but not the intervention or its implementation. Ethical permission was sought on these grounds.

## Conclusions

When evaluating service delivery or policy interventions, researchers may not have control over design and implementation of the programme or selection of sites. Where evaluation can be decoupled from the programme/intervention, clarity is needed about how to define and allocate responsibility. Requiring researchers to seek approvals for aspects of a programme that they have not initiated and do not control may obstruct, interfere with or prevent important research and learning without providing any further protections to research participants. We recommend that the Ottawa declaration, trial registration processes and other relevant guidelines should be reviewed as a matter of some urgency to address this challenge.
